# Visuomotor adaptation enhances representational acuity without altering spatial bias

**DOI:** 10.3389/fnhum.2025.1666476

**Published:** 2025-11-27

**Authors:** Carine Michel-Colent, Sarah Amoura, Olivier White

**Affiliations:** Université Bourgogne Europe, INSERM, CAPS UMR 1093, Dijon, France

**Keywords:** visuomotor rotation, space representation, line bisection, explicit and implicit learning, sensorimotor adaptation

## Abstract

**Introduction:**

Prism adaptation is a well-established paradigm for studying sensorimotor plasticity, known to produce not only motor after-effects but also changes in spatial cognition. Whether visuomotor rotation—a similar form of sensorimotor adaptation—elicits comparable cognitive transfer remains unclear.

**Methods:**

Participants performed visuomotor rotation tasks involving either leftward or rightward 15° rotations. The perturbation was introduced either abruptly (within one trial) or gradually (over 34 trials). To assess potential cognitive transfer, participants completed a perceptual line bisection task before and after adaptation.

**Results:**

No condition (leftward/rightward or abrupt/gradual) induced measurable cognitive after-effects in line bisection performance, indicating an absence of transfer from sensorimotor to spatial-cognitive domains. However, a novel finding emerged: visuomotor rotation enhanced participants’ representational acuity, reflected in improved sensitivity when judging the midpoint of a line. This effect was most pronounced following gradual perturbations and persisted beyond the adaptation phase.

**Discussion:**

These findings demonstrate a clear dissociation between the cognitive and perceptual consequences of visuomotor adaptation. Visuomotor rotation thus provides a reliable means to study sensorimotor plasticity without altering spatial representation—a methodological advantage for investigating populations with atypical spatial biases. The enhancement of representational acuity further suggests that sensorimotor learning can refine spatial discrimination independently of cognitive recalibration.

## Introduction

Humans tend to show a subtle but consistent attentional bias toward the left side of space—a phenomenon known as pseudoneglect. This bias is thought to arise from right-hemispheric dominance in visuospatial processing (Bowers and Heilman, 1980; Fink et al., 2001). It is typically measured using tasks like line bisection, where healthy individuals reliably err to the left of true center (Brooks et al., 2014; McCourt and Jewell, 1999). Importantly, pseudoneglect is not restricted to visual space; it extends to abstract representations that carry spatial associations, such as number magnitude (Loftus et al., 2009; Longo and Lourenco, 2007), letter order (Zorzi et al., 2006) and sound frequency (Michel et al., 2019).

Sensorimotor adaptation has proven to be a powerful tool to modulate these cognitive biases. In particular, short-term adaptation to leftward-shifting prisms can reverse pseudoneglect, inducing a rightward bias that mimics patterns seen in patients with spatial neglect (Fortis et al., 2011; Michel, 2006, 2016). These effects are not limited to perceptual space but also extend to symbolic domains, such as number lines and letter sequences (Loftus et al., 2008; Nicholls et al., 2008). Prism adaptation has therefore been widely used not only as a model for spatial cognition but also as a rehabilitation method for neglect (Jacquin-Courtois et al., 2013; Rossetti et al., 1998).

Recent studies have begun to disentangle the learning processes underlying these adaptation effects. Traditional frameworks emphasized recalibration (a fast correction based on error feedback) and realignment (a slower process aligning sensory reference frames). More recently, models distinguish between explicit and implicit learning instead. Explicit learning involves conscious strategy use and immediate error correction, whereas implicit learning refers to gradual, unconscious changes in internal models and sensorimotor mappings (O’Shea et al., 2014; Taylor et al., 2014). Crucially, only realignment appears to be associated with cognitive after-effects such as changes in space representation.

Yet, not all forms of sensorimotor adaptation produce these effects. For instance, adapting to a novel force field that perturbs limb dynamics alters motor behavior but does not typically shift spatial cognition (Leclere et al., 2019; Michel et al., 2018). This suggests that the type of sensorimotor conflict, and the adaptive mechanisms it engages, are critical in driving representational change. In this study, we revisited visuomotor rotation—a paradigm closer to prism exposure than force field adaptation. We manipulated both the direction of perturbation (leftward vs. rightward) and its mode of introduction (abrupt vs. gradual). Gradual exposure is known to promote slow and implicit behavioral change due to realignment and may therefore be more likely to induce cognitive after-effects (Jakobson and Goodale, 1989; Michel et al., 2007b). We hypothesized that only gradual adaptation to a leftward rotation would reverse pseudoneglect.

## Results

We designed an experiment to test if the transfer of sensorimotor effects induced by visual rotation to spatial representation is a phenomenon peculiar to prism adaptation. Participants were exposed to either leftward (L) or rightward (R) visuomotor rotations during reaching movements. The perturbation was introduced abruptly (ABR) or gradually (GRA). Different participants were assigned to four groups following a factorial design (G_*L,ABR*_, G_*L,GRA*_, G_*R,ABR*_, or G_*R,GRA*_). Participants performed a line bisection test before and after the adaptation phase.

### Groups adapted to gradual and abrupt visuomotor rotations

**FIGURE 1:**
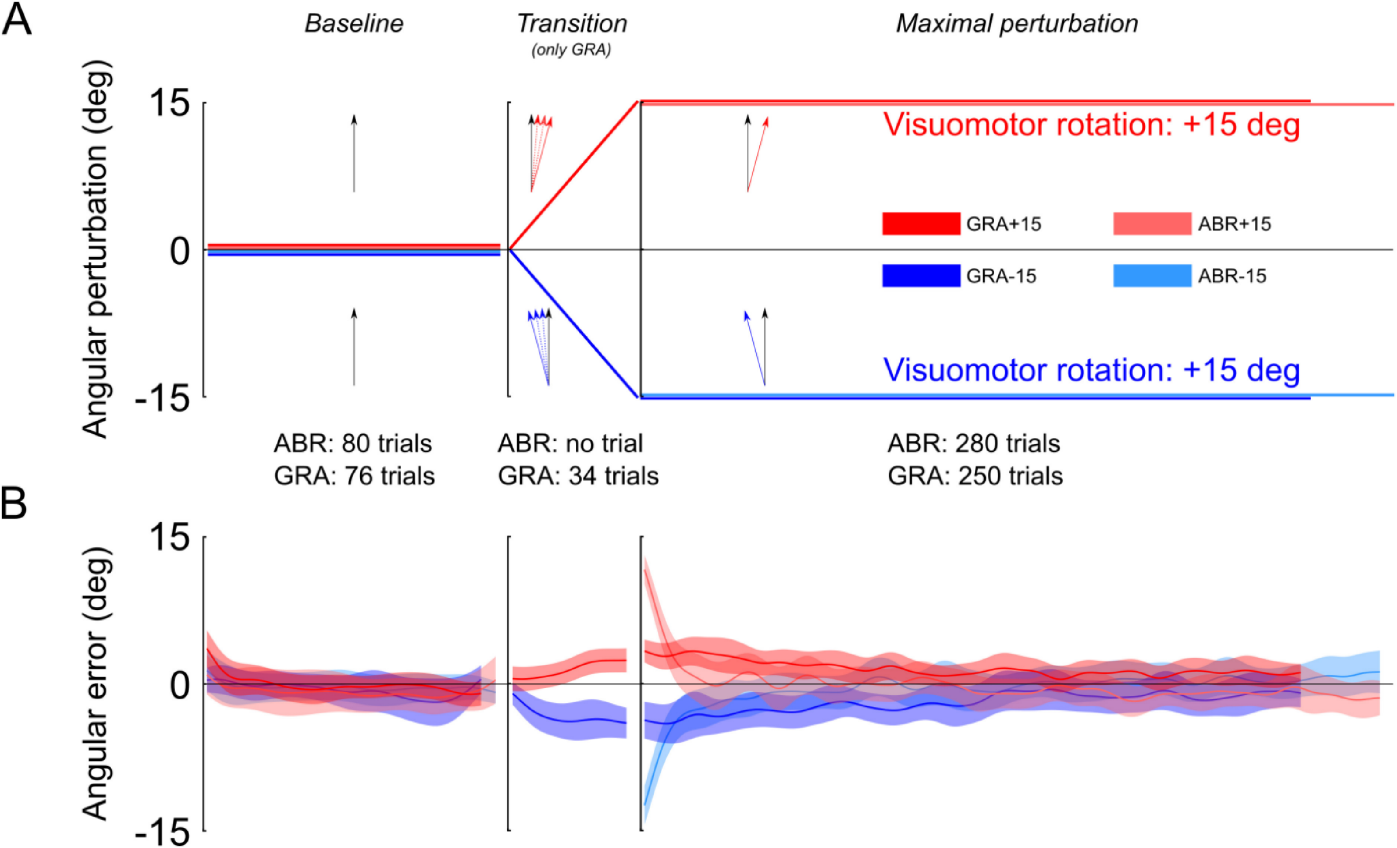
Illustration of the protocol and angular errors during the adaptation phase. **(A)** Participants in both groups were trained with pointing movements without visuomotor rotation (Baseline). During the transition, the GRA group was exposed to leftward or rightward visuomotor rotations that linearly ramped from 0 deg to either –15 deg or +15 deg (transition) and then reached a maximal perturbation for the remaining trials (Maximal perturbation). After the baseline, participants from the ABR groups were immediately exposed to maximal perturbations, without transition. **(B)** Mean and standard error of angular errors measured during the baseline, the transition (for the GRA group only) and the maximal perturbation phases. Notice the high and mirror symmetric errors in the first trials of the Maximal perturbation phase for the ABR group and moderate but still mirror symmetric errors in the GRA group.

Each group experienced a baseline sequence during which 80 trials were performed to one of five targets (Figure 1B, “Baseline”). No rotations were introduced in that sequence. The errors were on average −0.3 deg (SD = 1.4 deg). Within each group, the mean error was not different from 0 deg (independent *t*-test, G_*L,ABR*_: t_26_ = 0.06, *p* = 0.950; G_*L,GRA*_: t_26_ = −1.89, *p* = 0.071, G_*R,ABR*_: t_26_ = −1.20, *p* = 0.243 and G_*R,GRA*_: 1.36, *p* = 0.187) and group performances were comparable in terms of direction errors (all *t* < 0.28, all *p* > 0.272).

Two groups were exposed to abrupt perturbations (G_*L,ABR*_ and G_*R,ABR*_) and two groups experienced gradual perturbations (G_*L,GRA*_ and G_*R,GRA*_). Participants in the two ABR groups (left and right perturbations) exhibited large errors during the first trials in the “Maximal perturbation” sequence (Figure 1B, light colors). These errors were −12.9 deg in G_*L,ABR*_ and 13.5 deg in the G_*R,ABR*_. They reached similar absolute amplitude (t_26_ = −0.65, *p* = 0.520) and were different from 0 (G_*L,ABR*_: t_26_ = 16.80, *p* < 0.001, η^2^ = 0.92 and G_*R,ABR*_: t_26_ = 21.75, *p* < 0.001, η^2^ = 0.95). Participants in the GRA group also showed errors in the early trials of the “Maximal perturbation” sequence (Figure 1B, dark colors; G_*L,GRA*_: −4.8 deg and G_*R,GRA*_: 3.7 deg). However, their amplitudes, although the same in absolute values (t_26_ = 0.78, *p* = 0.443), were smaller than in the ABR groups (t_54_ = 10.61, *p* < 0.001, η^2^ = 0.68) and also different from 0 deg (G_*L,GRA*_: t_26_ = 4.01, *p* < 0.001, η^2^ = 0.39 and G_*R,GRA*_: t_26_ = 5.06, *p* < 0.001, η^2^ = 0.51). In contrast to the ABR groups, the GRA groups were exposed to a 34-trial transition during which the rotation of the cursor linearly went from 0 deg to −15 deg for G_*L,GRA*_ or +15 deg for G_*R,GRA*_ (Figure 1B, “Transition”). Unlike in the ABR groups, participants of the GRA groups did not report having detected any sensorimotor disturbance while using the robot. Statistics did not reveal significant differences between the last trials in the “Transition” sequence and the errors in the first trials of the “Maximal perturbation” sequence (G_*L,GRA*_: t_13_ = 0.91, *p* = 0.381, η^2^ = 0.39 and G_*R,GRA*_: t_13_ = 1.33, *p* = 0.206).

At the end of the long “Maximal perturbation” sequence, all groups were adapted to the visual rotation. Indeed, the errors during the last 3 trials were on average 0.03 deg (SD = 2.54 deg) and were not significantly different from 0 deg (all *t* < 1.9, all *p* > 0.710).

### Effects of sensorimotor adaptation on space representation

**FIGURE 2:**
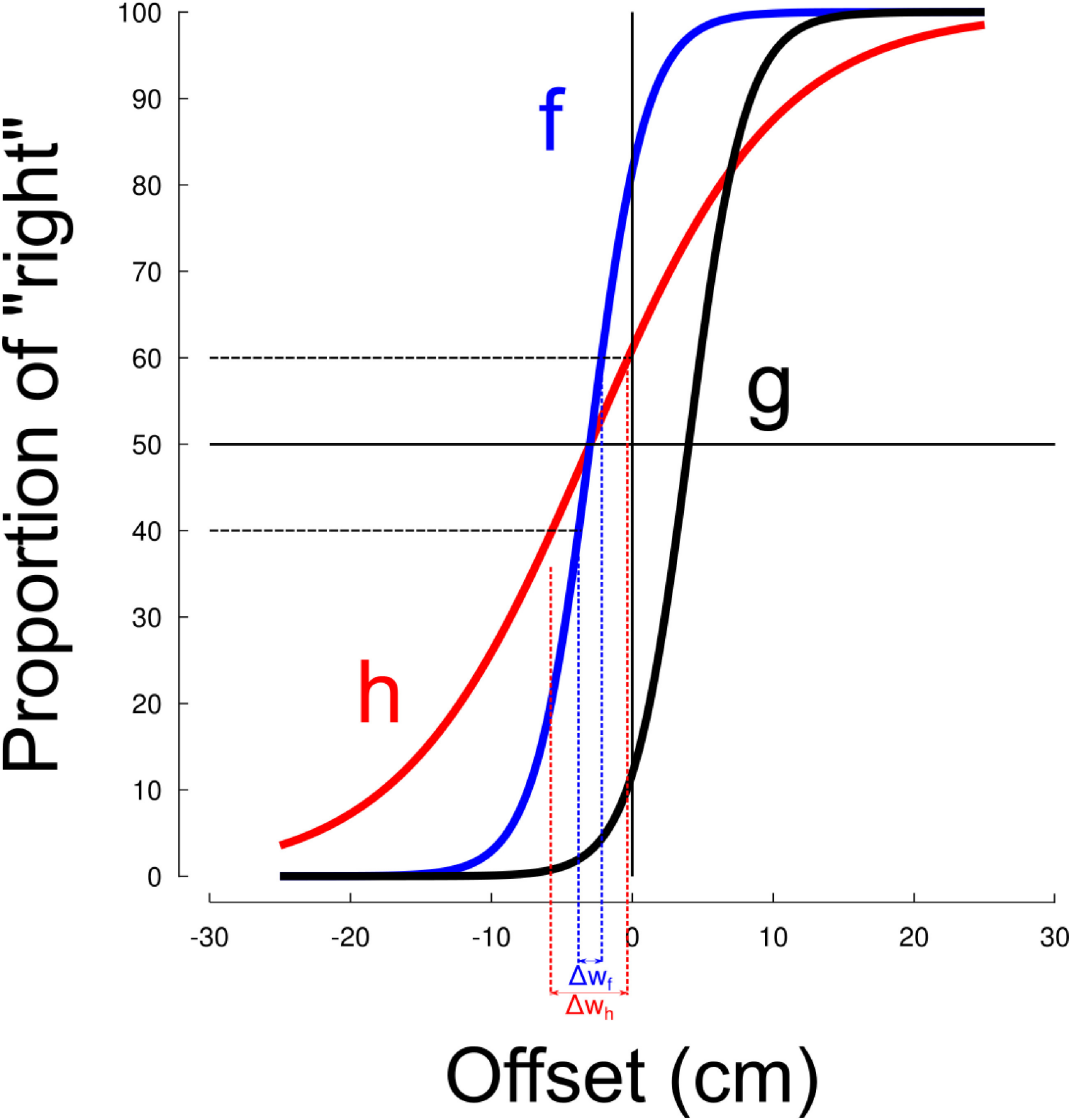
Subjective line center in the group exposed to abrupt (ABR) and gradual (GRA) perturbations. Blue colors correspond to leftward cursor rotations (–15 deg) and red colors correspond to rightward cursor rotations (+15 deg). Subjective line center before and after the adaptation phase are denoted by light and dark colors, respectively. No conditions affected this value.

We quantified the effect of visuomotor adaptation on space representation by means of a standard line bisection task. Figure 2 depicts subjective line center values regressed through the sigmoid model before (“Pre,” light colors) and after (“Post,” dark colors) sensorimotor adaptation separately in the ABR groups and GRA groups. In addition, the series are presented separately for the left (−15, blue) and right (+15, red) perturbation conditions. Positive estimation of line center values indicate a rightward perceived midpoint (rightward bias), negative values indicate a leftward perceived midpoint (pseudoneglect-like). Together, the four groups had initial rightward (0.16) estimation of line center (all different from 0, t_94_ = 4.7, *p* < 0.001, η^2^ = 0.19). This pattern was consistent at the group level (all *t* > 2.1, *p* < 0.045) except for the ABR+15 group (t_22_ = 1.9, *p* = 0.076, η^2^ = 0.14). All groups were, however, comparable (all *t* < 0.4, *p* > 0.656) before entering the visuomotor adaptation task (baseline checks are descriptive; *p*-values unadjusted). In our sample, the grand-mean pre-test subjective line center was slightly rightward, consistent with reports that baseline bias magnitude varies with viewing constraints and stimuli.

We made a statistical analysis to quantify the effects observed in Figure 2. We ran a RM-ANOVA with the factors modality (ABR vs. GRA), direction of perturbation (Left vs. Right) and Time (Pre vs. Post). None of these factors influenced space representation (all *F* < 0.5, all *p* > 0.474) and no interaction was significant either (all *F* < 1.3, all *p* > 0.255). Across participants, the subjective line center did not change reliably from Pre to Post mean difference = −0.0266, *t*(55) = −1.17, *p* = 0.247, dz = −0.16, 95% CI [−0.072, 0.019], Holm-adjusted. The absolute bias magnitude likewise showed no reliable change [Δ|subjective line center| = −0.0099, *t*(55) = −0.47, *p* = 0.641].

We then attempted to highlight any difference that would emerge with time within the Post period. The subjective line center was stable across the five Post blocks, F_4,163_ = 0.70, *p* = 0.608.

**FIGURE 3:**
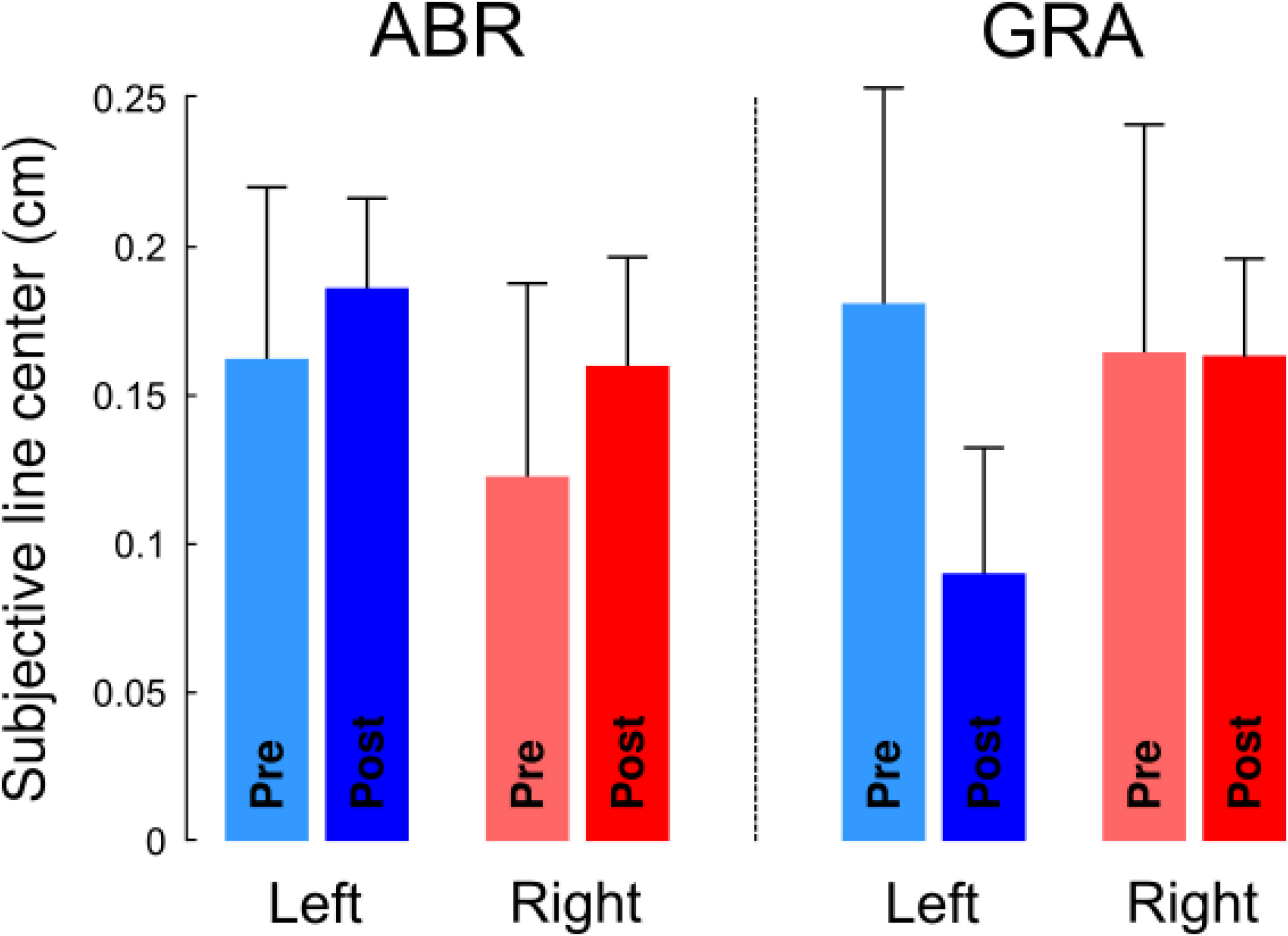
The width of the interval in which participants provide reliable responses to the line bisection task varies with modality (ABR, Abrupt and GRA, Gradual) and time but not direction of perturbation (L, Left vs. R, Right). The narrower the interval, the better the representational acuity. The *x*-axis of the right panel (Time) corresponds to the pre-adaptation block (ABR and GRA not different) and the five post-adaptation blocks. Representational acuity improves (Δw decreases) after exposure to visuomotor rotations and remains stable after. ns, not significant, **p < 0.01 (Holm-adjusted).

A measurement scale is defined not only by its graduations but also by its sensitivity to the phenomenon it measures. In this context, while our ability to perceive the position of a mark on a line segment may remain unchanged across experimental conditions, the sensitivity of the perceptual system itself could still be affected. In other words, experimental manipulations may not shift perceived position but could blur or enhance the clarity with which we discern the midline. To assess this, we tested how representational acuity (Δ*w*, see “Materials and Methods”) was influenced by experimental conditions. Figure 3 depicts Δ*w* in function of direction, modality and time. The RM ANOVA with factors Direction, Modality and Time reported a main effect of modality (F_1,111_ = 7.1, *p* = 0.009, η^2^ = 0.05) and time (F_4,111_ = 4.9, *p* = 0.0004, η^2^ = 0.16) on Δ*w* but not direction (F_1,111_ = 2.1, *p* = 0.155). *Post hoc* inspection revealed that Δw was smaller after gradual than abrupt exposure, indicating greater representational acuity under gradual perturbation (Holm-adjusted *p* < 0.05). Descriptively, the Post means were GRA = 0.10 ± 0.02 vs. ABR = 0.14 ± 0.02 (see Figure 3, right panel). Pre-test Δw did not differ across groups. Together, the Modality and Time effects show that gradual, implicitly weighted adaptation enhances perceptual precision beyond mere repetition. Furthermore, Δw dropped (acuity increased) immediately from Pre to the first Post block (all pairwise *t* > 2.54, Holm-adjusted *p* < 0.014) and then remained flat across Post blocks (all ∣ *t*∣ < 0.5, Holm-adjusted *p* > 0.619). There was no interaction between factors (all *F* < 1.74, all *p* > 0.189) and the four groups had comparable Δ*w* before adaptation (all *t* < 1.162, all *p* > 0.261).

To examine whether the improvement in representational acuity could be explained by task repetition or general alertness effects, we analyzed data from the control experiment. Reaction times did not significantly change from pre- to post-test [*t*(9) = 1.48, *p* = 0.17], indicating no reliable alertness-related facilitation in the absence of visuomotor perturbation. Representational acuity was stable across the five post-test blocks (all *p* > 0.5), but pooling across blocks revealed a modest improvement from pre to post [Pre: 0.160 ± 0.016; Post: 0.108 ± 0.021; *t*(9) = 2.51, *p* = 0.034, dz ≈ 0.79]. Thus, task repetition alone enhanced acuity to some extent, but the additional gains seen in the main experiment—particularly after gradual exposure—are not explained by practice effects alone.

## Discussion and conclusion

The main objective of the present experiment was to investigate whether adaptation to a visuomotor rotation transfers to spatial representation in healthy individuals. Despite using conditions that were optimal for promoting sensorimotor adaptation, and a sensitive paradigm for assessing spatial representation, we did not observe any representational after-effects. This absence of effect cannot be attributed to insufficient adaptation, as we used a prolonged exposure protocol and deliberately omitted a washout phase in order to maintain participants in an adapted state at the time of post-testing. However, our results revealed a consistent improvement in the sensitivity with which participants discriminated the center of the line following the perturbation.

Understanding the mechanisms underlying sensorimotor plasticity is crucial to identify the conditions that favor cognitive after-effects. As reviewed in the introduction, prism adaptation reliably produces after-effects on spatial representation in healthy individuals (Michel, 2016), whereas visuomotor rotation, as used in the present study, does not. Cognitive after-effects are thought to depend on the presence and strength of sensorimotor after-effects (Michel and Cruz, 2015). Therefore, understanding the mechanisms that enable sensorimotor adaptation to generalize beyond the immediate context of the perturbation is key to understanding its potential cognitive consequences. Note that our goal was not to replicate the prism adaptation literature, which is already well established, but rather to test whether a visuomotor adaptation protocol could produce similar effects.

Recent work (Fleury et al., 2019) has offered valuable insights into this issue by comparing sensorimotor processes across different paradigms. A first distinction lies in the relative contribution of explicit and implicit learning processes. When a motor command is issued, the forward model uses an efference copy to predict the sensory consequences of the movement. The mismatch between actual and predicted reafferent signals (prediction error) triggers adaptive processes aimed at preserving accuracy (Kawato, 1999; Wolpert and Miall, 1996). In early exposure phases, large prediction errors primarily engage explicit learning mechanisms, which use feedback to correct ongoing actions. These processes are fast and cognitively demanding but contribute minimally to after-effects. In contrast, implicit learning—a slower, more automatic process—realigns sensory and motor reference frames over time and is considered the main driver of after-effects (O’Shea et al., 2014). We hypothesized that a key difference between prism and visuomotor adaptation lies in the dominance of explicit versus implicit processes. Visuomotor adaptation may be largely governed by explicit strategies, which result in weaker and less generalizable sensorimotor after-effects (Ghahramani et al., 1996; Pine et al., 1996), and hence, minimal cognitive consequences. To counteract this, we used a gradual perturbation protocol—previously shown to promote implicit learning (Jakobson and Goodale, 1989; Kagerer et al., 1997; Michel et al., 2007b)—but even under these favorable conditions, no representational after-effects were observed.

A second important consideration concerns the context in which these adaptive processes unfold. While prism and visuomotor rotations both induce visual-proprioceptive mismatches, they differ fundamentally in their reafferent signals. In prism adaptation, these signals are direct, body-centered, and interface-free, leading to broad, context-independent adjustments. In contrast, visuomotor rotations involve symbolic, indirect reafferences via a robotic device and computer display. In this case, actions are not body-centered, and the interface introduces context dependencies (Fleury et al., 2019; Kluzik et al., 2008). Moreover, visuomotor rotations are centered on the start position of the movement, whereas prism-induced shifts operate in eye-centered coordinates—an important distinction that may affect generalization. The concept of generalization refers to the persistence of adaptive changes across different contexts (Bastian, 2008; Censor, 2013; Poggio and Bizzi, 2004), and depends on the nature of the exposure. While prism adaptation leads to robust generalization and context-independent changes (Bedford, 1989; Fleury et al., 2019), visuomotor rotation shows poor generalization, with narrow spatial tuning and a steep gradient around the trained direction (Ghahramani et al., 1996; Pine et al., 1996). This likely explains why no transfer to broader spatial, cross-modal, or representational contexts was observed in our line bisection task. This interpretation is consistent with recent findings showing that perceptual consequences of visuomotor adaptation tend to remain spatially limited to the trained movement directions (Rand and Heuer, 2019).

The absence of representational after-effects following visuomotor rotation is an important finding with methodological implications. Like force field adaptation (Michel et al., 2018), visuomotor rotation appears to be an effective tool for studying sensorimotor plasticity without influencing spatial representation, as measured with line bisection. This makes visuomotor rotation a particularly useful paradigm for investigating adaptation in populations that exhibit baseline spatial biases (e.g., hyper pseudoneglect in schizophrenia or reversed pseudoneglect in children with dyslexia) (Michel et al., 2007a,^2011^), as it allows for assessment of motor plasticity without worsening representational imbalances. It should also be noted that participants in our sample did not show a strong pseudoneglect bias at baseline, which may have limited the potential to detect any leftward shifts following leftward perturbation.

Another more novel finding from this study was the improvement in the precision with which participants judged the center of the lines—a feature we refer to as representational acuity. Representational acuity improved after exposure to visuomotor rotation, and this improvement was slightly enhanced by gradual exposure. This concept aligns with the previously described “indifference zone,” the portion of the line perceived as centered (Manning et al., 1990; Nicholls and Roberts, 2002), and is known to depend on stimulus characteristics (Westheimer et al., 2001). Analogous findings exist in the somatosensory domain, where perceptual acuity improves following active or passively guided reaching tasks (Bernardi et al., 2015). In our study, this enhancement in visual representational acuity persisted after adaptation, with no evidence of reversal over time. While this effect may reflect a practice effect, its amplification in the gradual group—despite identical pre/post exposure durations across conditions—suggests a potential contribution of sensorimotor adaptation. Importantly, the Modality main effect on Δw demonstrates that gradual exposure—favoring implicit realignment—sharpens representational acuity more than abrupt exposure. This supports the view that implicit sensorimotor recalibration contributes to durable improvements in perceptual precision.

The control experiment provides further context for interpreting these results. General improvements in acuity occurred with task repetition alone, consistent with a practice-related contribution. However, only visuomotor adaptation involving active error correction produced a reliable decrease in reaction times (alertness effect) and stronger gains in representational acuity, particularly under gradual exposure. This suggests a dual contribution to post-adaptation improvements: a baseline learning effect due to repetition, and an additional enhancement driven by sensorimotor correction demands. Importantly, the critical contrast central to our study—the greater representational acuity improvement following gradual relative to abrupt adaptation—cannot be explained by repetition or alertness alone.

These results highlight a close link between sensorimotor learning and perceptual precision. It is likely that exposure to intersensory conflict—even indirectly, as in visuomotor rotation—enhances the sensitivity of the sensory modalities engaged in the adaptive process. In this case, the ability to discriminate the center of a line was sharpened. More broadly, our findings reveal a dissociation between cognitive after-effects on spatial representation (which did not occur) and after-effects on representational acuity (which improved) following visuomotor rotation.

In conclusion, this study presents two key findings with both theoretical and methodological significance. First, under the specific conditions we used, visuomotor rotation can serve as an effective tool to study sensorimotor plasticity while sparing representational space. Second, exposure to visuomotor rotation enhances representational acuity. Future work using varied experimental conditions will help further characterize how different forms of sensorimotor adaptation influence both spatial representation and perceptual precision.

This study has a few limitations that should be acknowledged. First, no measurement of proprioceptive or sensorimotor after-effects was conducted at the end of the experiment. While our design sought to maintain participants in an adapted state during post-testing, the absence of these measurements prevents a direct assessment of the persistence of adaptation effects. Second, the sample size per group (*n* = 14) was relatively modest for a between-subject design. Although the main comparison was conducted within subjects (pre- vs. post-exposure), this may limit the statistical power to detect small effects. Finally, participants did not consistently exhibit a strong pseudoneglect bias prior to adaptation, potentially reducing the likelihood of observing shifts in spatial representation. Despite these limitations, our study offers new insight into the differential effects of visuomotor adaptation on spatial cognition and perceptual acuity.

## Materials and methods

### Participants

Fifty-six right-handed adults (26 women, mean age = 23 years old, SD = 4.8) voluntarily participated in the main experiment and ten other participants were enrolled in the control experiment (4 women, mean age = 22.7 years old, SD = 1.8). All participants were healthy, without neuromuscular disease and with normal or corrected to normal vision. All participants gave their informed consent prior to their inclusion in the study, which was carried out in agreement with legal requirements and international norms (Declaration of Helsinki, 1964). The procedures of this observational study were approved by the local ethics committee of Université Bourgogne Europe. All participants were naïve as to the purpose of the experiments and were debriefed after the experimental sessions.

### Experimental procedure and apparatus

The experiment consisted of three distinct phases: BISECTION-PRE, ADAPTATION, and BISECTION-POST, described in detail below. In brief, we assessed spatial representation using a line bisection task conducted before and after a visuomotor adaptation protocol involving passing through targets with a haptic device.

Throughout the entire experiment, participants were comfortably seated in front of a virtual environment in a dimly lit room, with their heads stabilized on a chin and forehead rest. Reaching movements were performed with a Phantom Premium 3.0 robotic arm (SensAble Technologies, USA) operating in endpoint position-control mode. Handle position (X–Y) was read directly from the robot’s encoders at 500 Hz, and instantaneous velocity and acceleration were inferred numerically from these encoder signals (finite difference algorithm). The on-screen cursor was rendered from robot-space coordinates ensuring precise spatial correspondence between physical and visual spaces. Visual stimuli were displayed on two 24-inch LCD screens (1920 × 1080 px, 60 Hz) arranged in a calibrated stereo-mirror setup—left eye viewing the left screen, right eye the right screen—creating a stereoscopic image aligned with the workspace. The visuomotor rotation was applied in software as an angular deviation of the cursor trajectory relative to the start position, with a render latency < 2 ms. Spatial alignment between robot and visual space was verified before each session by a calibration procedure (maximum positional error < 1 mm).

During the BISECTION-PRE phase, participants performed a line bisection task. They viewed 130 horizontal green segments (400 mm long, 2 mm thick), each presented with a perpendicular red tick (30 mm tall, 2 mm thick). In a forced-choice format, participants judged whether the tick marked a point to the left or to the right of the segment’s true center. Responses were provided verbally and manually recorded by the same experimenter. If no response was given within 20 s, the trial was recorded as missing.

Tick offsets followed a Gaussian distribution centered on the Euclidean midpoint of the segment, with increased sampling density near the center to enhance the sensitivity of subsequent analyses. To prevent participants from developing a conscious strategy, they were explicitly informed that the tick would never appear exactly at the midpoint, and that its distribution would not be systematically symmetric. Trials were presented in random order, and a blank white screen was shown for 1,500 ms between each trial to eliminate visual cues and prevent carryover effects from one trial to the next.

We conducted a technical validation experiment to confirm that our line bisection task was sensitive to well-established spatial cues. To this end, we recruited an additional 13 participants (9 women; mean age = 23.6 years, SD = 4), who performed the same bisection task used in the main experiment. Each participant completed a total of 234 trials, presented in three blocks of 78 trials. In this validation task, a blue circle appeared on either the left or right side of the segment in one-third of the trials each (33%). In the remaining third of trials, no visual cue was presented. The three cue conditions (left, right, none) were randomized across trials to prevent predictability. We found that the presence of the left cue significantly shifted the perceived center of the segment to the left compared to when there were no cues (paired *t*-test: t_12_ = 2.3, *p* = 0.032, $\eta_{p}^{2}=0.21$). We found the same effect, but reversed, when the right cue was displayed (paired *t*-test: t_12_ = 2.5, *p* = 0.022, $\eta_{p}^{2}=0.27$). Our setup then reliably replicates the known cueing effects on space representation (Milner et al., 1992). These cue-dependent subjective line center shifts provide construct validity for the forced-choice psychometric approach used here.

During the ADAPTATION phase, participants used a robotic arm to reach for targets from a fixed starting position, marked by a white circle (diameter: 0.3 cm). One of five circular targets (diameter: 0.3 cm) appeared on each trial, positioned along an invisible circle centered on the starting point with a radius of 20 cm. Targets were randomly presented either directly above the start position (0 deg), or at ± 10 deg and ± 20 deg relative to vertical (i.e., −20 deg, −10 deg, 0 deg, +10 deg, +20 deg). The real-time position of the robotic handle was visually represented by a blue cursor (diameter: 0.2 cm). A trial began when the cursor entered the starting circle. Participants were instructed and trained to reach the targets with a peak velocity between 65 and 75 cm/s. As feedback, the target turned red if the movement exceeded 75 cm/s, or blue if it was below 65 cm/s. After each trial, the robot gently guided the participant’s hand back to the starting position to avoid active return movements and prevent motor planning during repositioning.

Participants were randomly assigned to one of four experimental groups (*n* = 14 per group), based on the direction and type of visuomotor perturbation applied (Figure 1A). In the ABR+15 group, a rightward 15-deg cursor rotation was introduced abruptly after 80 baseline trials and maintained for the following 280 trials. The ABR-15 group experienced the same procedure, but with a leftward 15-deg rotation. In contrast, the GRA+15 and GRA-15 groups began with 76 baseline trials, after which the visuomotor rotation was introduced gradually over 34 trials. This transition was implemented as a linear increase in rotation by 0.429 deg per trial, eventually reaching either +15 deg (GRA+15) or −15 deg (GRA-15). Participants in these groups then completed 250 additional trials at the full rotation level.

All participants, regardless of group, completed a total of 360 trials. The cumulative visuomotor perturbation (in absolute degrees) reached 4,200 deg in the ABR groups and 4,005 deg in the GRA groups, representing a relative difference of only 4.6%. Importantly, we chose not to include a washout phase in order to preserve the participants’ adapted state for the post-adaptation assessments. Post-experiment debriefing revealed that participants in the abrupt (ABR) condition were aware of the visuomotor perturbation, whereas those in the gradual (GRA) condition remained unaware of any change.

During the BISECTION-POST phase, the device setup, task, and instructions were identical to those used in the BISECTION-PRE phase, with the only difference being the number of trials. In this phase, participants were presented with a total of 195 horizontal green segments, divided into five blocks of 39 trials each. As in the pre-test, the distribution of tick offsets within each block was carefully balanced. This design allowed us to assess potential effects of time on spatial representation across the course of the post-adaptation phase.

In addition, a separate control experiment was conducted with ten right-handed participants (4 women, mean age = 22.7 years, SD = 1.8). These participants completed the same sequence of pre-bisection, reaching, and post-bisection tasks as in the main experiment, except that no visuomotor rotation was applied during the reaching phase. This design allowed us to assess the potential contributions of general task repetition or exposure to the robotic setup, independent of visuomotor adaptation.

### Data analysis

In the BISECTION-PRE and BISECTION-POST phases, we recorded participants’ verbal responses for every trial (“Is the tick positioned to the right or to the left of the veridical segment midline?”). We then calculated the proportion of “RIGHT” responses as a function of the offset separately in each block (one block in BISECTION-PRE and 5 blocks in BISECTION-POST). This S-shaped function saturated at 0% for large negative (left) offsets and at 100% for large positive offsets (right).

To quantify the offset that corresponded to chance level (50%), i.e., the subjective perceptual estimation of the line center, we fitted logistic functions in each block, and for each participant separately (r^2^ = 0.91 on average), $f=\frac{100}{1+e^{-a_{2}\,\left(t-a_{1}\right)}}$, where a_1_ and a_2_ were regressed and *t* corresponds to the offset. The subjective line center that corresponds to f = 50 is a_1_.

**FIGURE 4:**
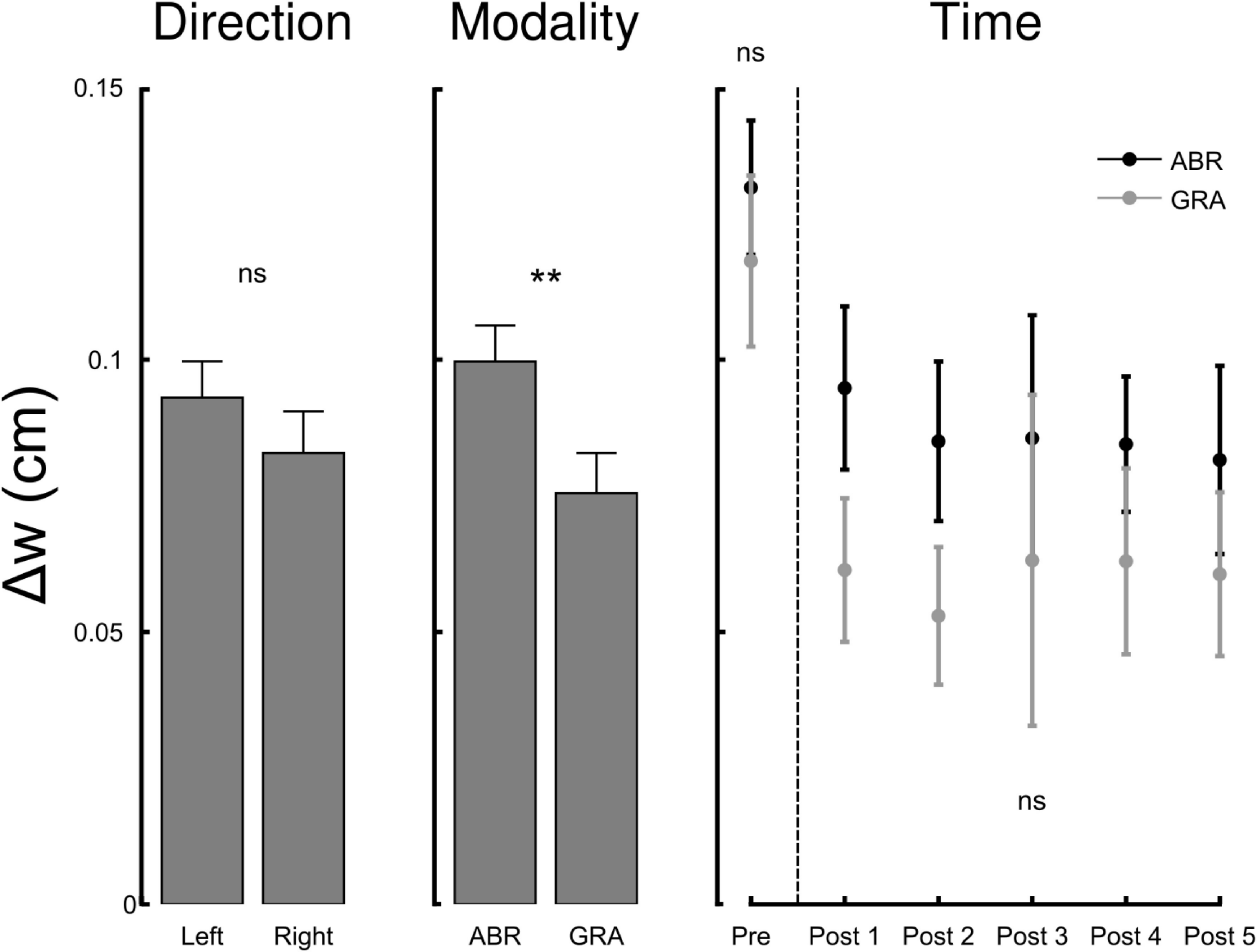
Simulation of logistic functions. Functions f and g are parallel (same slope) but have different offsets (−3 for f and +4 for g), thus yielding different subjective centers. Function h has the same offset as f but a smaller slope (reduced sensitivity). Functions were defined as $f=\frac{100}{1+e^{-0.5\,\left(t+3\right)}},g=\frac{100}{1+e^{-0.5\,\left(t-4\right)}},h=\frac{100}{1+e^{-0.15\,\left(t+3\right)}}$.

Restricting the analysis of the distributions of the responses only to the offset is too limitative. Indeed, different logistic regression functions can yield the same subjective line center. Figure 4 depicts three simulated examples of logistic regression lines (f, g and h). One can see that functions f and h intersect at exactly the ordinate of 50%, and therefore lead to the same offset (−3 cm). Function g has a different offset (4 cm). We therefore also quantified the sensitivity of the decision curve with the derivative of the model. We define the perceived representational acuity as Δ*w*. This parameter is calculated as the difference between two values of w, defined as $w=\frac{1}{a_{2}}\,\ln\,\left[\frac{p_{u}\,\left(100-p_{l}\right)}{p_{l}\,\left(100-p_{u}\right)}\right]$, with [*p*_*l*_; *p*_*u*_]=[40%; 60%] and Δ*w*=*w*_*u*_−*w*_*l*_. For a given probability of response (20% centered on 50%, or chance level), a small Δ*w* means that the regression curve is steep and that one can decide with high sensitivity (certainty) whether an offset is to the right or to the left of the center. In contrast, a large Δ*w* reflects a flatter regression curve and a low sensitivity (uncertainty) to discriminate between left and right ticks. In the extreme (and hypothetical) case of a perfectly flat regression line, responses would be entirely dictated by chance (in that case, Δ*w* = + ∞). Thus, the smaller Δ*w*, the greater the perceived representational acuity.

We used a 2-alternative forced-choice judgement of “tick to the left/right of true center,” and fit a logistic psychometric to obtain the subjective line center and slope-derived Δw (representational acuity). Relative to free bisection on a continuous ruler, forced-choice (i) isolates perceptual decision from motor end-point placement, and (ii) yields a bias (subjective line center) and a precision (Δw) estimate on the same trials. A separate validation showed the task is sensitive to exogenous spatial cues, shifting PSEs left/right as expected (see “Technical validation experiment”).

During the ADAPTATION phase, cursor positions were recorded with a sampling rate of 500 Hz. Movement start was detected when movement velocity exceeded 3 cm/s for at least 100 ms. Direction error of each movement was defined as the angle between straight ahead and the segment connecting the start position to the position of the cursor 150 ms after movement onset.

### Statistical analyses

All inferential analyses were implemented as repeated-measures ANOVAs for each dependent variable (subjective line center and Δw). The model included Time (Pre, Post) as a within-subject factor and Modality (ABR, GRA) and Direction (Left, Right) as between-subject factors. Planned pairwise Pre→Post comparisons within each Modality × Direction were used only to clarify significant model effects, and *p*-values were adjusted using the Holm–Bonferroni procedure within each endpoint. This ensured that all pairwise tests were nested within the primary model, maintaining a clear inferential hierarchy and control of the family-wise error rate. The control (no-adaptation) group was analyzed separately to estimate potential practice or alertness effects.

Quantile–quantile plots were used to verify the normality of residuals before applying parametric tests. Independent *t*-tests were conducted to compare data between groups, and paired *t*-tests were used for within-subject comparisons when appropriate. We report *F*, *p*, partial η^2^ (and 95 % CIs for effects/contrasts), and Cohen’s d or dz for pairwise contrasts. Manipulation checks during the adaptation phase and descriptive baseline comparisons were not adjusted. Sensitivity analyses (α = 0.05, power = 0.80) indicated that the Pre/Post paired comparison of the subjective line center was powered to detect effects of dz ≈ 0.38 or larger, and that the ABR–GRA contrast on Δw was powered to detect effects of d ≈ 0.76. All data processing, model fitting, and statistical analyses were performed using custom routines in MATLAB (The MathWorks, Chicago, IL).

## Data Availability

The raw data supporting the conclusions of this article will be made available by the authors, without undue reservation.
